# Remodeling of Mitochondria in Cancer and Other Diseases

**DOI:** 10.3390/ijms24097693

**Published:** 2023-04-22

**Authors:** Yong Teng

**Affiliations:** 1 Department of Hematology and Medical Oncology, Winship Cancer Institute, Emory University, School of Medicine, Atlanta, GA 30322, USA; yong.teng@emory.edu; Tel.: +1-(404)712-8514; 2 Wallace H. Coulter Department of Biomedical Engineering, Georgia Institute of Technology & Emory University, Atlanta, GA 30322, USA

Mitochondria are highly dynamic and responsive organelles capable of fission and fusion and are a hub of diverse signaling pathways that are fundamental to cellular homeostasis, energy production, metabolism, survival, and death [1,2,3]. Mitochondrial remodeling, including rearrangement, recycling, and reprogramming, is essential for mitochondrial quality control, structural integrity, and functional interaction with other cellular organelles. Although we are far from fully understanding the complexity of mitochondrial remodeling, it has been implicated in the pathogenesis of many human diseases, including cancer and age-related diseases. With the advent of new information, technologies, and methodologies, it is time to delineate more precisely and comprehensively the significance and enigmatic mechanisms relevant to dynamic mitochondrial remodeling in response to pathophysiological stress. These updated knowledge and provocative ideas are important for the effective translation of science into practice.

In this Special Issue, we have brought together several contributors to provide evidence-based insights into the mechanistic and pathogenic roles of mitochondrial remodeling and dynamic networks to support the discovery of novel therapeutic targets or new mitochondria-targeted therapies in order to cure mitochondria-associated human diseases. After a rigorous peer review process, eight articles were collected, consisting of three comprehensive reviews [4,5,6] and five original research articles [7,8,9,10,11]. Notably, these articles were contributed by renowned academic institutions from different countries engaged in mitochondria-based biology and translational research, demonstrating great interest in this trending area of research.

Although mitochondrial oncology has been known for well over 20 years, the field has experienced a resurgence of interest in the recent decade. As a newly discovered member of the mitochondrial AAA+ family, ATAD3A is essential for mitochondrial homeostasis, lipid metabolism, and communication with the endoplasmic reticulum. Our recent studies have demonstrated that elevated ATAD3A levels in several cancers (e.g., breast cancer and head and neck cancer) are closely associated with increased cancer cell proliferation, metastasis, and resistance to certain treatments [12,13]. In our review paper [4], we highlight the potential of ATAD3A as a target for the development of novel cancer therapies that could inhibit aberrant cancer metabolism and progression. There are many molecules (e.g., PKC, WASF3, and FAT1) and pathways that regulate ATAD3A expression and function. However, the multiple roles of ATAD3A reflect the complete precision required to recognize the various regulators and effector molecules of the protein. One of the important challenges is to understand how interactions between ATAD3A and other mitochondrial proteins and effectors are spatially and temporally regulated. The delineation of both the biological and pathological roles of ATAD3A will then allow us to shed light on the steps required to develop more effective anti-ATAD3A approaches for cancer treatment.

The tuning of mitochondria-associated processes under pathophysiological conditions is fundamental to the balance between cell death and survival. Mitochondria are essential players in cancer cell survival, as they are involved in processes ranging from metabolism to Ca^2+^ signaling and from the regulation of mitochondrial dynamics to oxidative stress. Chemoresistance, as a survival strategy adopted by cancer cells upon apoptotic stimulation, is inextricably linked to mitochondrion-related pathways. To this end, Genovese et al. critically evaluated current evidence on the relationship between mitochondria and cancer chemoresistance [6]. They conclude that the advances in the characterization of tumorigenesis and chemoresistance in the context of mitochondrial functionality are not only interesting but also crucial for the understanding of cancer mechanisms and the design of optimal therapeutics against uncontrolled cell survival and resistance to apoptotic stimuli.

Platelets isolated from peripheral blood are an accessible source of mitochondria that have been studied in various diseases. However, details on the mitochondrial bioenergetic functions of platelets in cancer patients are not available. Palacka’s group has shown reduced platelet mitochondrial metabolism and endogenous CoQ_10_ concentrations in non-hospitalized patients after 3–6 weeks of acute COVID-19 disease [7]. Therefore, they further tested whether platelet mitochondrial oxygen consumption and OXPHOS could be affected in chemotherapy-naïve patients with urothelial carcinoma [7]. This study provides new insights into the potential role of platelets in the pathogenesis of urothelial carcinoma, suggesting that increased oxidative stress, decreased OXPHOS, and reduced endogenous platelet CoQ_10_ levels may contribute to the reprogramming of platelet mitochondrial OXPHOS toward the activation of glycolysis in cancer cells. In addition, the impaired mitochondrial function may contribute to increased oxidative stress by initiating reverse electron transport from CoQ_10_ to complex I. 

Mitochondrial dysfunction has been implicated in a variety of diseases other than cancer [14]. For example, mitochondrial dysfunction has been implicated in neurodegenerative diseases (e.g., Alzheimer’s, Parkinson’s, and Huntington’s diseases) and metabolic disorders (e.g., diabetes, obesity, and metabolic syndrome). In addition, mitochondrial dysfunction has been linked to cardiovascular disease, respiratory disease, and aging. Nichenko and her colleagues reported the accumulation of damaged mitochondria due to insufficient autophagy in the pathophysiology of skeletal muscle aging [11]. Ulk1 is an autophagy-related kinase that initiates autophagosome assembly and plays a role in autophagosome degradation. To better understand the contribution of reduced autophagy to sarcopenia, they investigated the effects of a lifelong deficiency of Ulk1-mediated autophagy on aging skeletal muscle phenotypes. The team used highly sensitive electrophysiological techniques to assess muscle contractile and metabolic functions as well as changes in autophagy signaling and flux in aged mice with the genetic deletion of Ulk1 and their littermate controls. This study showed that Ulk1 plays a dual role in maintaining mitochondrial integrity by autophagosome assembly and degradation, supporting a function of Ulk1-mediated autophagy in aging skeletal muscle. Another study provides important evidence that mitochondria play a pathogenic and targetable role in developmentally programmed heart disease and that mitochondrial differences are not only exposure-related but also sex-specific [10]. These novel findings suggest that in addition to impaired bioenergetics, the process of converting nutrients into usable energy, oxidative stress, and accelerated cell death also plays a role in the progression of mitochondria-associated programmed cardiac disease under metabolic stress.

The articles included in this collection are intended to add value to the field by highlighting the potential of targeting mitochondria as a therapeutic approach for cancer and other diseases. This will inspire new potential strategies of new drugs or other interventions aimed at correcting mitochondrial defects and remodeling and mitigating their effects on disease development and progression. 
